# RadStat: An open-source statistical analysis tool for counts obtained by a GM counter

**DOI:** 10.1371/journal.pone.0267610

**Published:** 2022-05-31

**Authors:** Mehrdad Shahmohammadi Beni, Hiroshi Watabe, Wing Sum Kwan, M. Rafiqul Islam, Kwan Ngok Yu

**Affiliations:** 1 Department of Physics, City University of Hong Kong, Hong Kong, China; 2 Division of Radiation Protection and Safety Control, Cyclotron and Radioisotope Center, Tohoku University, Sendai, Japan; 3 Graduate School of Biomedical Engineering, Tohoku University, Sendai, Japan; Central State University & Ohio University, UNITED STATES

## Abstract

The interaction of ionizing radiation with matter is a stochastic process and statistical analysis of such a process would be a crucial step in understanding radioactivity. Geiger–Müller (GM) counter is a widely used radiation detector used in nuclear radiation surveying, which produces counts upon exposure to a radioactive source. There are a variety of multi-purpose software that can be used to perform statistical analysis of measured counts from a GM counter. However, statistical analysis is a lengthy, error prone and time-consuming process, which gets more tedious when the number of measurements increases. In the present work, we have developed an open-source and easy-to-use graphical user interface (GUI) computer program named RadStat for statistical analysis of counts measured by a GM counter. RadStat has its own scripting syntaxes and bundled with gnuplot for quick visualization of output results. We believe the present open-source GUI program would be a useful tool for research and teaching of nuclear radiation physics.

## Introduction

Nuclear radiation is ubiquitous in our daily life, in that we are exposed to radiation on a daily basis from various sources [1–5]. The interaction of radiation with matter is a stochastic process, which means statistical analysis of the process would be a crucial step in understanding the underlying physics of such interactions [6, 7]. For example, Geiger–Müller (GM) counter which is one of the most commonly used detectors for surveying nuclear radiation [8–11], provides information on the count or count rate upon exposure to a radiation source. The produced counts would be meaningless on their own and statistical analysis would be required to provide physical meanings to these count readings. The basic information was provided by the mean counts with their associated standard deviations that estimated the most probable count and the corresponding spread of the data, respectively.

Once the counts from a radiation source are obtained, it is useful to check the distribution of the counts against Poisson distribution by first putting the counts into fixed sized bins (i.e., performing binning) to ensure that the experimental measurements have been properly performed. In addition, it is also common to approximate the Poisson distribution with the normal distribution using the mean counts and the standard deviation [12, 13]. The average count is obtained from the measured experimental counts and then the standard error would be calculated according to the sample size. Individual count readings can be checked against 1σ ($\bar{x}\pm \surd \bar{x}$) and 2σ ($\bar{x}\pm 2\surd \bar{x}$) limits [14].

The abovementioned statistical analysis is a lengthy, error prone and time-consuming process, which gets more tedious when the number of measurements increases. To the best of our knowledge, there is no dedicated open-source and easy-to-use software that is tailor-made for statistical analysis of GM counter data, and most researchers and students use tools such as Microsoft Excel, MATLAB, and other multi-purpose software to carry out statistical analysis of their experimental data. In the present work, we have developed an open-source graphical user interface (GUI) program named RadStat for statistical analysis of GM counter data. RadStat has its own specific scripting language to perform user defined statistical analysis. RadStat has been bundled with gnuplot (http://www.gnuplot.info/) for ease of plotting and visualization of the results. There are variety of tools and software that can perform general statistical analysis such as SPSS Statistics [15], SAS/STAT [16], Stata [17], Minitab [18] and many other packages. These software packages are for general statistical analysis and not dedicated to statistics of nuclear radiation. In addition, there are other tools that were developed for statistical analysis of nuclear radiation. For example, ROOT [19] is a powerful software framework written in C++ programming language that provides statistical analysis, data processing and visualization, however users need to learn and use C++ programming language to communicate with the program. The ADAQ framework [20] uses C++ and Python libraries and has been designed to streamline the acquisition and analysis of radiation detector data produced in digital data acquisition (DAQ) systems and in Monte Carlo detector simulations; this software mainly focuses on data acquisition and lacks in-depth statistical analysis functions. InterSpec [21] is a native or web application to assist in analyzing spectral nuclear radiation data, using a peak-based methodology; this software would be useful in identifying radionuclides, source age and activity, shielding amounts and dose rate calculations by feeding the obtained spectrum into the software. In comparison, RadStat is easy to use, since no programming knowledge would be required, and the statistical features provided by RadStat would be useful for early learners in this field to verify the statistics involved in nuclear radiation measurements.

Radioactivity and interaction of radiation with matter is a stochastic process. The variations in the recorded counts from a radioactive source is the result of this stochasticity. It would be important to understand the extent of this variation and the count that best represents the activity of the radioactive sample. Theoretically the measured counts from a radioactive source followed the Poisson distribution, which under most conditions can be approximated using the normal distribution. The obtained raw data (i.e., counts) would be meaningless without statistical analysis. For example, the noise corresponding to statistical fluctuation in the counts obtained through nuclear radiation measurements could easily lead to false positives or false negatives if the data have not been rigorously checked using proper statistics. The statistical analysis tools offered by RadStat program would be useful to further verify the statistics involved in nuclear radiation measurements. We believe the present program would be useful for statistical analysis of GM counter data and can be particularly useful and beneficial for teaching and learning activities for young researchers and students in the field of nuclear radiation physics.

## Material and methods

RadStat was written in the FORTRAN90 programming language. Microsoft PowerStation QuickWin run-time libraries and Microsoft Fortran libraries (using MSFLIB) were used in building the GUI [22, 23]. The FORTRAN90 programming language was chosen mainly due to its fast computational speed and numerical stability. In addition, the use FORTRAN90 programming language would make the task easier to bundle RadStat with Monte Carlo packages such as Monte Carlo N-Particle (MCNP) and Particle and Heavy Ion Transport code System (PHITS) radiation transport codes that were written in FORTRAN programming language. In addition, gnuplot uses Qt5 libraries for plotting the output data. RadStat is a portable computer program that does not require installation and it has a size on disk of ~492 KB. In the present work, we have developed our own syntaxes for a variety of widely used statistical functions. This makes statistical analysis easier to perform, and can thus facilitate pedagogical applications. Using these scripts, the users do not need to write their own computer programs, which can be relieving to those users who are not good at computer programming. These scripting syntaxes are summarized in Table 1.

**Table 1:** List of RadStat syntaxes and their specific functions.

Syntax	Function	Syntax	Function
Banner	*RadStat banner*	mean	*Calculates mean*
timedate	*Time and date*	stdev	*Calculates standard deviation*
info	*Developer information*	stdevpois	*Calculates standard deviation from Poisson distribution*
radstatdef	*RadStat project*	stdevdiff	*Calculates difference between standard deviations*
meandef	*Mean definition*	min	*Minimum value finder*
stdevdef	*Standard deviation definition*	max	*Maximum value finder*
normappx	*Normal approximation*	bin	*Binning the data*
samplestat	*Comment on sampling*	lowbin	*Smallest bin*
siglimcheck	*Sigma limit check*	highbin	*Largest bin*
distbdiff	*Difference in distributions*	norm	*Normal distribution estimation*
sample	*Sampling the data*	pois	*Poisson distribution estimation*
1siglim	*1-sigma analysis*	2siglim	*2-sigma analysis*
gmtip	*Some GM counter tips*		

**Fig 1:**
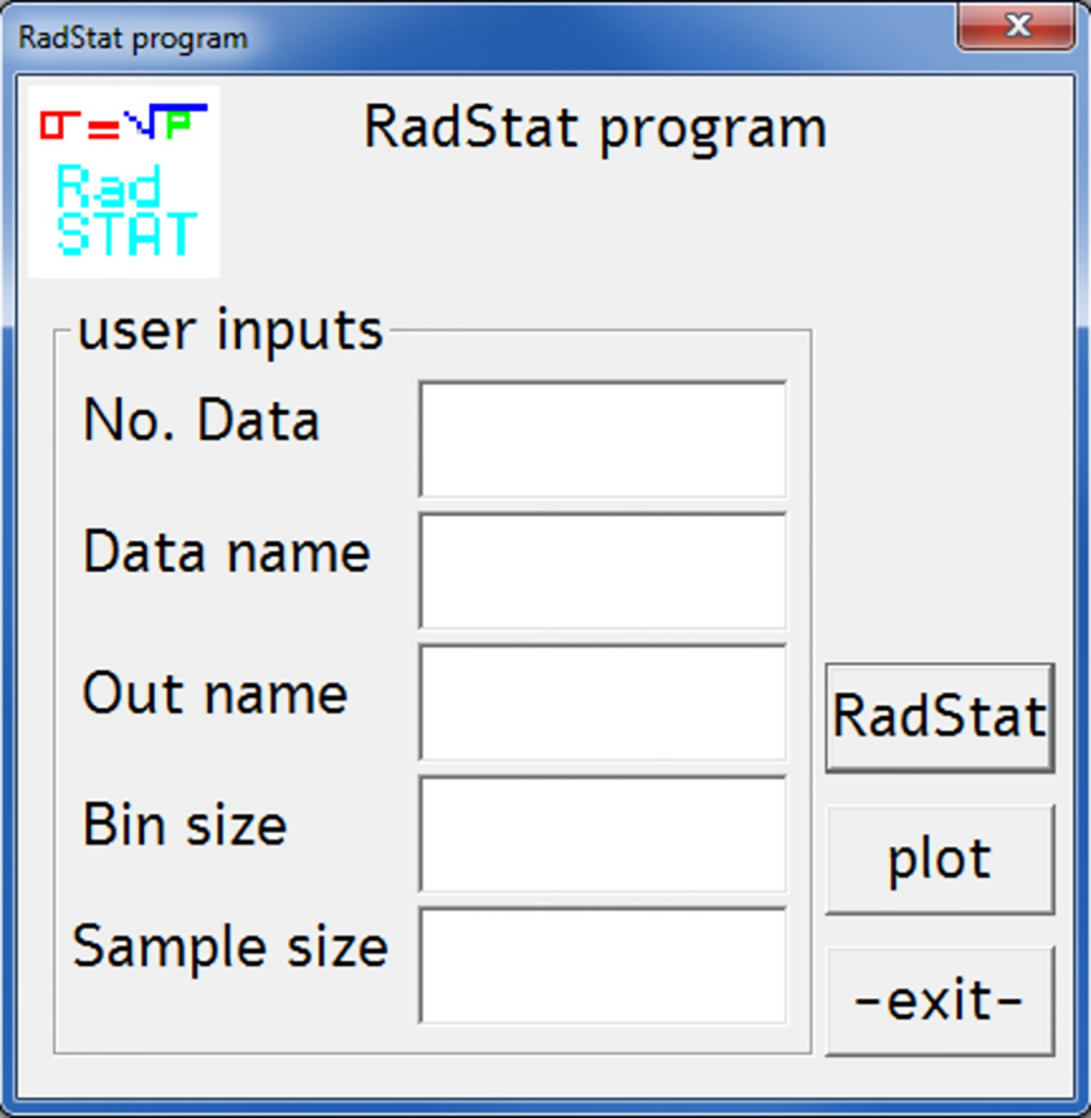
Snapshot of RadStat GUI computer program.

The syntaxes programmed into RadStat should be written in a dedicated script data file for the program to read. The user can use any of the commands shown in Table 1 to perform a specific statistical analysis. These scripting syntaxes would be read as string and then detected by the program after reading the script data file. In addition to these scripting syntaxes, the GUI would also take some inputs such as raw data number, raw data name, output file name, bin and sample size, which are shown in Fig 1.

These GUI inputs were read in the form of 256-character lengths and were then converted to floating point 4-byte real numbers. RadStat has been bundled with gnuplot (http://www.gnuplot.info) to plot the binned counts from the experimental measurements and to provide theoretical counts using Poisson and normal distributions. Estimation from the Poisson distribution was calculated as

$$p_{pois}(x)=\frac{e^{-\lambda }\times \lambda^{x}}{x!}$$ (1)

where *p*_*pois*_*(x)* was the probability getting the value *x* from the Poisson distribution with the mean value *λ*. It is remarked that for large *x* values it would be tedious to numerically calculate the factorial of *x* as needed in Eq (1). In order to circumvent this, we used the Stirling’s approximation [24, 25] to numerically compute the factorial. The method provided a close approximation to the computed probability from the Poisson distribution. The Poisson distribution could also be approximated using the normal distribution as

$$p_{norm}(x)=\frac{1}{\sigma \surd 2\pi }e^{-\frac{1}{2}{\left(\frac{x-\mu }{\sigma }\right)}^{2}}$$ (2)

where *p*_*norm*_*(x)* was the probability from the normal distribution of *x*, *μ* was the mean and *σ* was the standard deviation. The use of gnuplot in combination with RadStat would make the comparison much easier through graphical representation. The program outputs the plotting data in gnuplot format after the plot button has been clicked.

The fluctuations arising from the counting statistics represent an unavoidable source of uncertainty in all nuclear radiation measurements; these fluctuations can be quantified and compared with the predictions from statistical distributions such as Poisson and normal distribution [26]. Previously, Tsoulfanidis and Landsberger [27] provided a detailed description on the statistical analysis process in nuclear radiation measurements and provided all the required steps; these were implemented in the RadStat computer program. To the best of our knowledge, there is no software like RadStat computer program which is tailor-made for statistical analysis of counts from nuclear radiation measurements that has the ease of use, functionality and its own syntaxes.

The RadStat program and its source code could be downloaded from https://figshare.com/articles/software/RadStat_An_open-source_statistical_analysis_tool_for_counts_obtained_by_a_GM_counter/17876057. The current version of our program is executable on Microsoft Windows systems. In addition, we have tested the present program in GNU/Linux environment using Wine and it worked well. RadStat does not require any installation and it can run in portable mode. RadStat is distributed under GNU General Public License version 3 (GNU GPLv3). We have used measurements from a ^90^Sr radioactive source to test the present computer program. A total of 100 measurements each obtained within 10 second-intervals were measured using the ST360 GM counter system. The GM tube voltage was set at 840 V and the total counts (source + background radiation) were measured. If our objective was to determine the “net count” (i.e., “gross count”–“background count”) due to the source itself, the “background count” should be subtracted, in particular for relatively weak sources. However, the objective of the example here was to study the distribution of the gross count, which followed the Poisson distribution, so the “background count” was not subtracted.

The RadStat program cannot analyze the data in real-time; this is mainly due to the fact that entire count arrays must be read by the program and specific array size must be allocated to the memory prior to runtime. The data from the ST360 counter was recorded and transferred to the computer manually. The source activity was 407 Bq and the source to detector distance was 2 cm.

## Results and discussion

The 100 measured counts are shown in Table 2. These counts were used as input data to test our RadStat program. All commands shown in Table 1 were used to demonstrate the capability of RadStat. Three different cases were chosen for this numerical test, namely, (1) bin size of 5 and sample size of 10, (2) bin size of 10 and sample size of 20 and (3) bin size of 2 and sample size of 5.

**Table 2:** The 100 measured counts each obtained within 10 second-intervals using a GM counter.

172	171	177	196	168	199	161	182	158	185
183	190	183	180	194	206	176	185	189	175
191	197	187	180	149	179	179	180	176	168
166	157	196	181	161	186	175	211	184	176
177	158	166	186	157	191	168	194	199	183
171	211	194	182	168	199	190	172	181	177
163	181	176	200	177	174	179	176	188	165
179	197	181	186	182	171	173	166	170	191
174	183	169	192	170	184	179	180	181	172
165	167	178	180	155	159	164	180	191	170

The direct output of statistical analysis from RadStat for the measured data is shown in Box 1. In this analysis, bin size of 5 and sample size of 10 were used. All commands shown in Table 1 were used to demonstrate the full capability of RadStat. The output from RadStat provides the users with all required statistical analyses for the measured experimental data. The RadStat program produces Google search in an automatic manner, which helps the users to get more information on each topic as necessary.

**Box 1. Statistical analysis output from RadStat program for bin size of 5 and sample size of 10**

1. Analysis of the measured data in file by RadStat
2. Data file name: data100.dat
****** ****** ****** ****** ****** ****** ******
* * * * * * * ** * * **
****** ****** * * ****** ** ****** **
* * * * * * ****** ** * * **
* * * * * * * ** * * **
* * * * ****** ****** ** * * **
---The RadStat project---
10. We were inspired to develop this project and computer program after teaching nuclear radiation labs for many years at City University of Hong Kong. The present tool and scripting language would be useful to familiarize the students and young researchers with the concept of statistics of nuclear radiation. The program aims at analysis of GM counter data. RadStat loads in the measured experimental data and performs the required statistical analysis.
---Some GM counter tips---
12. A Geiger counter (Geiger-Muller tube) is a device used for the detection and measurement of all types of ionizing radiation: alpha, beta and gamma radiation. Basically, it consists of a pair of electrodes surrounded by a gas. The electrodes have a high voltage across them. The gas used is usually Helium or Argon. When radiation enters the tube, it can ionize the gas. The ions (and electrons) are attracted to the electrodes and an electric current is produced. A scaler counts the current pulses, and one obtains a “count” whenever radiation ionizes the gas. The apparatus consists of two parts, the tube and the (counter + power supply). The Geiger-Mueller tube is usually cylindrical, with a wire down the center. The (counter + power supply) have voltage controls and timer options. A high voltage is established across the cylinder and the wire as shown on the page of figures. When ionizing radiation such as an alpha, beta or gamma particle enters the tube, it can ionize some of the gas molecules in the tube. From these ionized atoms, an electron is knocked out of the atom, and the remaining atom is positively charged. The high voltage in the tube produces an electric field inside the tube. The electrons that were knocked out of the atom are attracted to the positive electrode, and the positively charged ions are attracted to the negative electrode. This produces a pulse of current in the wires connecting the electrodes, and this pulse is counted. After the pulse is counted, the charged ions become neutralized, and the Geiger counter is ready to record. another pulse. In order for the Geiger counter tube to restore itself quickly to its original state after radiation has entered, a gas is added to the tube [28]. In conclusion, the Geiger-Muller counter is cool;) copy the link into your browser for a quick google search:
13. https://www.google.com/search?q=geiger+muller+counter
---How data mean/average is calculated?---
15. mean: sum of a collection of numbers divided by the count of numbers in the collection [29]. Here, the collection refers to the inputted measured data into the program. Simply, sum all the data points and divide it by the total number of data points. copy the link into your browser for a quick google search:
16. https://www.google.com/search?q=arithmetic+mean
---How data standard deviation is calculated?---
18. standard deviation: measure of the amount of variation or dispersion of a set of values [30]. Here, the set of values are those measured data inputted to the program. Mean of data will be needed in order to calculate the standard deviation. copy the link into your browser for a quick google search:
19. https://www.google.com/search?q=standard+deviation
---Normal approximation to Poisson distribution---
21. For sufficiently large values of mean, (say mean>1,000), the Normal Distribution is an excellent approximation to the Poisson Distribution. If mean is greater than about 10, then the Normal Distribution is a good approximation if an appropriate continuity correction is performed [31]. copy the link into your browser for a quick google search:
22. https://www.google.com/search?q=normal+approximation+to+poisson+distribution
*******info*******
24. RadStat program developed by Ben, report any issues or bugs
25. to: ben.sh@my.cityu.edu.hk
26. Time, date, zone
27. 2021     11     19     540     22     34     0     0
28. mean/average:         179.010
29. standard deviation from data:         12.321
30. standard deviation from Poisson distb.:         13.379
31. difference of standard deviations:         1.058
32. smallest value in measured data array:         149.000
33. largest value in measured data array:         211.000
34. smallest bin value:         147.000
35. largest bin value:         211.000
36. 147.000     151.000     1.000     1.000     1.162     1.232
37. 152.000     156.000     2.000     1.000     2.605     2.634
38. 157.000     161.000     9.000     7.000     4.981     4.906
39. 162.000     166.000     16.000     7.000     8.159     7.957
40. 167.000     171.000     28.000     12.000     11.502     11.242
41. 172.000     176.000     41.000     13.000     14.017     13.833
42. 177.000     181.000     62.000     21.000     14.827     14.826
43. 182.000     186.000     76.000     14.000     13.664     13.840
44. 187.000     191.000     85.000     9.000     11.013     11.254
45. 192.000     196.000     91.000     6.000     7.789     7.970
46. 197.000     201.000     97.000     6.000     4.850     4.917
47. 202.000     206.000     98.000     1.000     2.667     2.642
48. 207.000     211.000     100.000     2.000     1.299     1.236
*******Poisson-Normal*******
50. 147.000     151.000     -.070
51. 152.000     156.000     -.029
52. 157.000     161.000     .075
53. 162.000     166.000     .202
54. 167.000     171.000     .261
55. 172.000     176.000     .185
56. 177.000     181.000     .001
57. 182.000     186.000     -.176
58. 187.000     191.000     -.241
59. 192.000     196.000     -.182
60. 197.000     201.000     -.067
61. 202.000     206.000     .025
62. 207.000     211.000     .063
63. The normal distribution approximation to the Poisson distribution produces fully/partially smaller estimated count values compared to those from Poisson distribution.
64. The one-sigma (one standard deviation) is:         69.000%
65. 31.000% of data falls outside one-sigma (one standard deviation)
*****one-sigma (one standard deviation)*****
67. One standard deviation, or one-sigma define a region that includes 68 percent of all the data points
68. The two-sigma (two standard deviation) is:         96.000%
69. 4.000% of data falls outside two-sigma (two standard deviation)
*****two-sigma (two standard deviation)*****
71. Two standard deviation, or two-sigma define a region that includes 95 percent of all the data points
*******Sampling*******
73. sample size:         10
  1 174.100
  2 181.200
  3 180.700
  4 186.300
  5 168.100
  6 184.800
  7 174.400
  8 182.600
  9 181.700
  10 176.200
*******Sample analysis*******
75. sample mean:         179.010
76. sample variance:         28.673
77. sample standard deviation:         5.355
78. sample standard deviation from mean:         4.231
---end of file---

**Fig 2:**
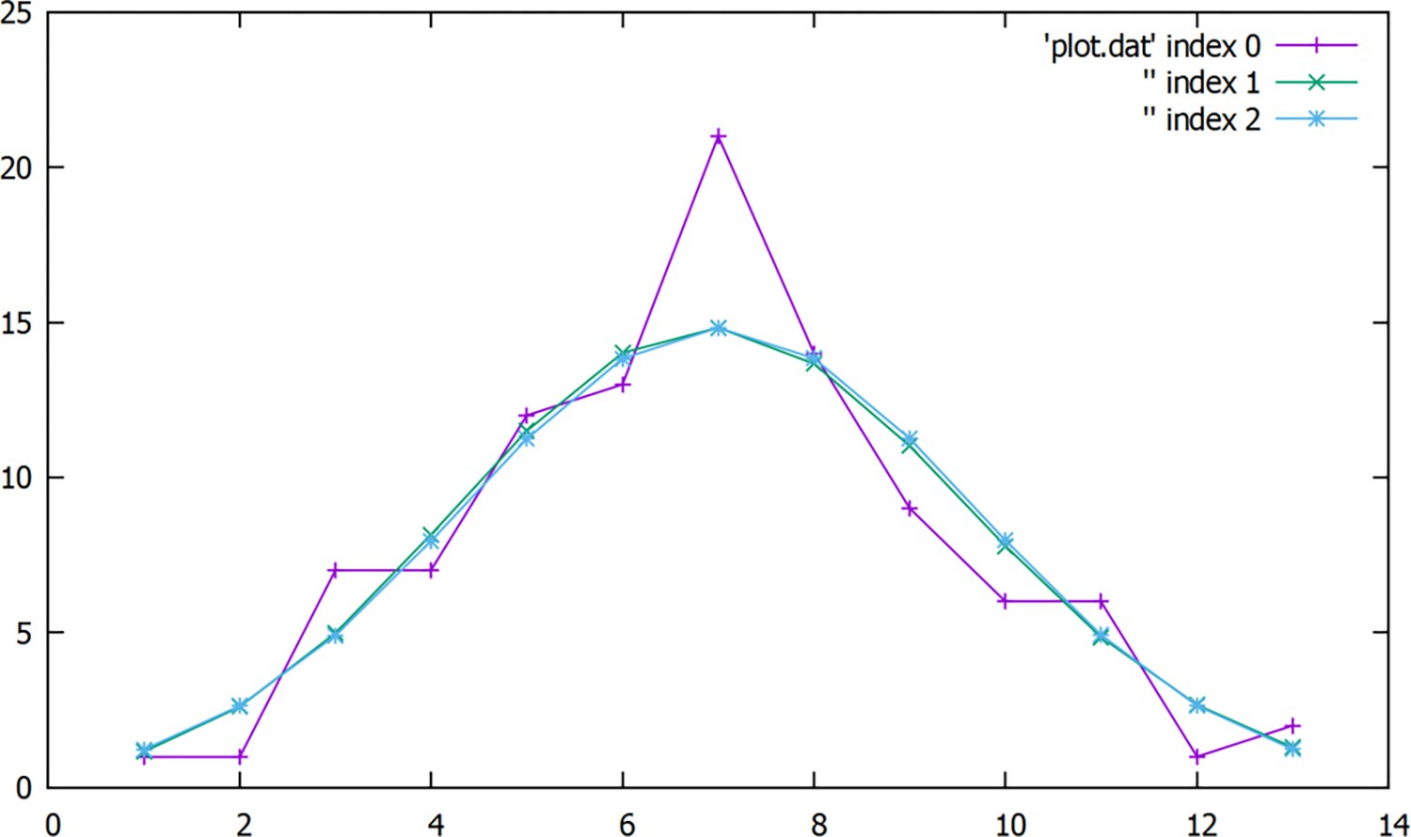
Gnuplot of binned results outputted by RadStat from experiment (purple), Poisson estimation (green) and normal approximation (blue). The x and y-axis represent the bin number and counts in each bin, respectively.

The experimental results were binned with a size of 5 (lines 36 to 48 in Box 1). The bins, cumulative number, number in each bin, estimation from Poisson distribution and approximation with normal distribution are summarized in Table 3. The estimations from Poisson and normal distributions for the number count in each bin were close to those measured experimentally. Since the total number of experimental measurements was 100, the cumulative counts added up to 100. The obtained results in Table 3 were plotted using gnuplot (that was bundled with the RadStat program) and shown in Fig 2.

**Table 3:** Binning of measured data with estimations from Poisson distribution and normal distribution approximation.

Low bin	High bin	Cumulative	Number	Poisson	Normal
147	151	1	1	1.162	1.232
152	156	2	1	2.605	2.634
157	161	9	7	4.981	4.906
162	166	16	7	8.159	7.957
167	171	28	12	11.502	11.24
172	176	41	13	14.017	13.83
177	181	62	21	14.827	14.83
182	186	76	14	13.664	13.84
187	191	85	9	11.013	11.25
192	196	91	6	7.789	7.970
197	201	97	6	4.850	4.917
202	206	98	1	2.667	2.642
207	211	100	2	1.299	1.236

The difference between the Poisson and normal distribution estimations are also shown in the output of RadStat presented in Box 1 (lines 49 to 62). In addition, based on the obtained differences, the program automatically comments on the obtained differences (line 63). In addition, in Box 1 (lines 64 to 71), the 1σ ($\bar{x}\pm \surd \bar{x}$) and 2σ ($\bar{x}\pm 2\surd \bar{x}$) limits were used to check the percentage of data that fell inside or outside the 1σ and 2σ limits. Furthermore, the results of sampling and sample analysis (sample size set to 10 in this test) are shown as output results (lines 72 to 78) in Box 1. Several different test cases were performed using RadStat with different bins and sample sizes. The output results for these tests obtained from RadStat can be downloaded from: https://figshare.com/articles/software/RadStat_An_open-source_statistical_analysis_tool_for_counts_obtained_by_a_GM_counter/17876057.

## Conclusions

In the present work, an open-source GUI computer program named RadStat was developed. RadStat is dedicated to performing full statistical analysis of experimentally data obtained using a GM counter, but it can also be used to analyze data obtained using other radiation detectors. RadStat reads the input data in ascii format and provides binning and sampling functions based on user-defined bin size and sample size. RadStat has its own scripting language, which is simple to use and understand. The program estimates the counts in each bin using Poisson distribution and approximates these using the normal distribution. In addition, checks against 1σ ($\bar{x}\pm \surd \bar{x}$) and 2σ ($\bar{x}\pm 2\surd \bar{x}$) limits would be performed and the program automatically comments on the results from this check. Finally, the program performs sampling and sample analysis of the user-inputted data. RadStat is a portable computer program which does not require installation. RadStat was bundled with gnuplot to enable quick visualization of the output results. We believe the present open-source GUI program would be a useful tool for research and teaching of nuclear radiation physics and in general radiation science. Recently, we have developed the MCHP (Monte Carlo + Human Phantom) platform to facilitate teaching nuclear radiation physics using MCNP Monte Carlo package [32]. In future works, we aim to bundle RadStat into the MCHP platform; this would be particularly useful to perform statistical analysis of nuclear radiation interaction with human body and organs. Such statistical analysis would help enhance the understanding on the significance of interaction events from different ionizing radiations with human body and organs.

## Supporting information

S1 Fig Comparison between RadStat and Microsoft Excel in estimating probability of occurrence from Poisson distribution (for λ = 1, 4, 10, 50, 70 and 90). In order to test the success of the present program in estimating Poisson probabilities, we have performed comparison between the results (for λ = 1, 4, 10, 50, 70 and 90) generated from RadStat and those from Microsoft Excel version 16.0.12527.22079 using the POISSON built-in function. These were tested for different cases and solved from 1 to 100 number of occurrences. From the comparison shown in Fig 1, good agreement was obtained between the estimated results from RadStat and those from Microsoft Excel.
(TIF)
