# Supplementary figures and images for: RadStat: An open-source statistical analysis tool for counts obtained by a GM counter
Source: PLoS One. 2022 May 31;17(5):e0267610. doi: 10.1371/journal.pone.0267610 (PMC9154119; doi:10.1371/journal.pone.0267610)

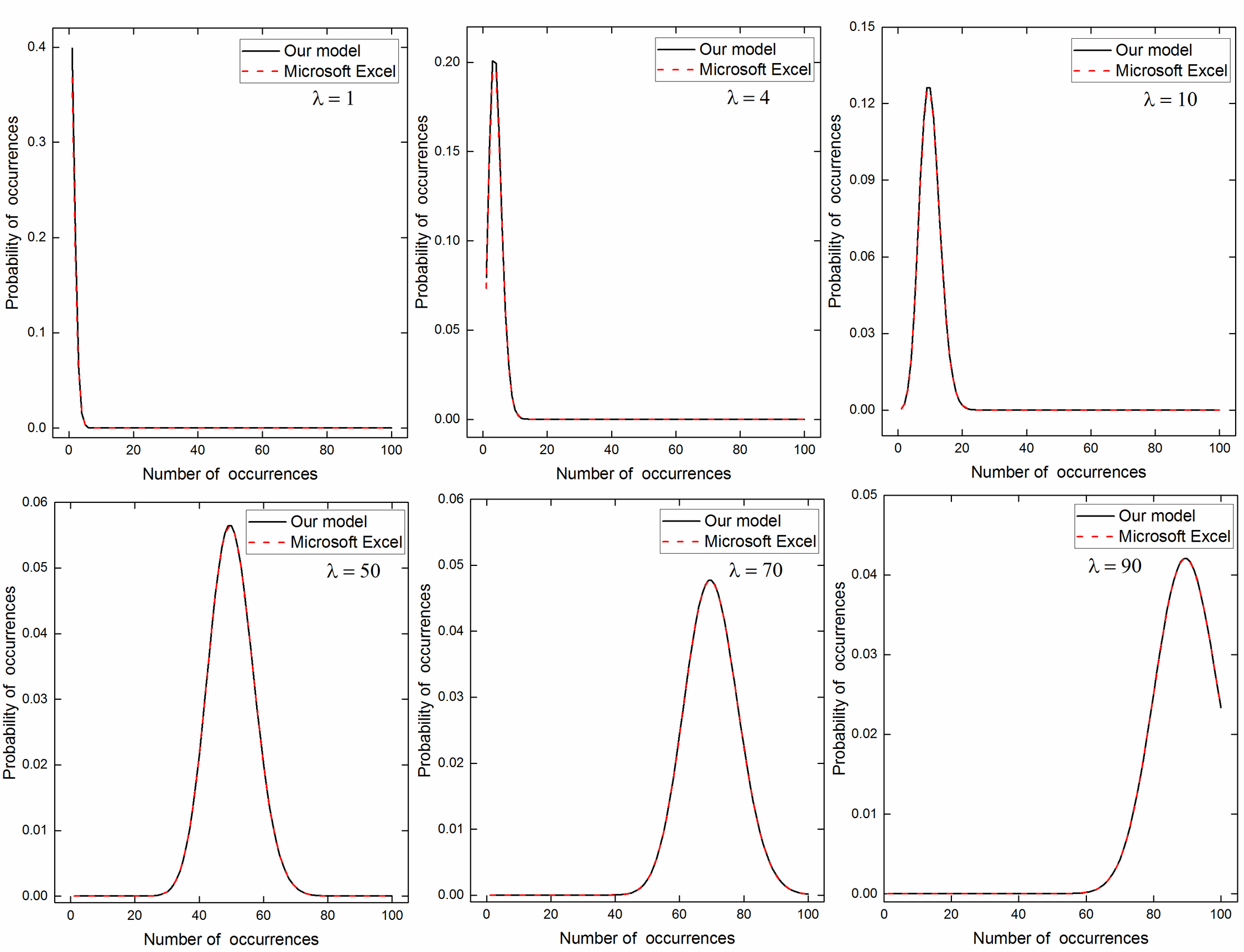

Supplement: S1 Fig — In order to test the success of the present program in estimating Poisson probabilities, we have performed comparison between the results (for λ = 1, 4, 10, 50, 70 and 90) generated from RadStat and those from Microsoft Excel version 16.0.12527.22079 using the POISSON built-in function. These were tested for different cases and solved from 1 to 100 number of occurrences. From the comparison shown in Fig 1, good agreement was obtained between the estimated results from RadStat and those from Microsoft Excel. (TIF) [file pone.0267610.s001.tif]
